# Editorial: Approaches to Advance Cancer Vaccines to Clinical Utility

**DOI:** 10.3389/fimmu.2019.02032

**Published:** 2019-08-27

**Authors:** An M. T. Van Nuffel, Caroline Boudousquié, Sandra Tuyaerts

**Affiliations:** ^1^The Anticancer Fund, Brussels, Belgium; ^2^Department of Oncology, Centre Hospitalier Universitaire Vaudois, Lausanne, Switzerland; ^3^Division of Gynecologic Oncology, Department of Oncology, KU Leuven, Leuven, Belgium; ^4^Leuven Cancer Institute (LKI), Leuven, Belgium

**Keywords:** cancer vaccines, personalized, adjuvant, antigen-presenting cell, *in situ* vaccination, immunosuppression, biomarkers

Although cancer vaccines have yielded promising results both *in vitro* and in animal models, their translation into clinical application has not been very successful so far, even though encouraging results from small early phase trials are reported. Junco et al. describes the 10-year follow up of Heberprovac, a GnRH1 peptide vaccine linked to a tetanic toxoid epitope in prostate cancer patients. Kjeldsen et al. reports on the 6-year follow up of an indoleamine-2,3-dioxygenase (IDO) peptide vaccine in non-small cell lung cancer. Both vaccines target endogenous proteins, are tolerated well long-term, and are safe and show durable responses. Delivering durable benefits is a unique feature of immune therapy, hence the emergence as “Breakthrough of the Year” 2013 (1). Through the success of immune checkpoint inhibitors, the tumor immunotherapy field revived and led to important new insights. A better understanding of the functional capacity of different dendritic cell (DC) subsets and the immunogenicity of tumor antigens, more particularly of neoantigens, have important implications for the improvement of cancer vaccines. These insights can guide the development of novel strategies, to enhance the clinical utility of cancer vaccines. The aim of this Research Topic was therefore to provide a comprehensive overview of current issues regarding cancer vaccine development with an emphasis on novel approaches toward enhancing their efficacy.

Current cancer treatments are becoming more and more **personalized** based on the patient's specific tumor characteristics instead of a one-size-fits-all approach (2). This concept is also true for cancer immunotherapies. Mastelic-Gavillet et al. describes personalized dendritic cell (DC)-based vaccination and mentions the importance of targeting private tumor antigens, such as **neoantigens**. Related to this, Klausen et al. discuss the use of alternative neoantigens resulting from JAK2 and CALR mutations in hematological malignancies. They also depict the use of regulatory proteins, PD-L1 and PD-L2, as target antigens. This latter is conceptually similar to the IDO vaccine trial described by Kjeldsen et al. as the immune target does not need to identify the tumor, but focuses on the suppressive environment. In the trial of Junco et al., the chosen target is a driver of tumor growth. Vermaelen discusses the recent efforts taken to improve the selection of tumor antigens to use as targets in cancer vaccines and their visibility. Xiang et al. identifies the most optimal peptide for vaccination from three antigens expressed by gynecological tumors.

An important issue to consider when aiming to increase the efficacy of cancer vaccines is the use of the right **adjuvant** (3). Besides using DC's as nature's adjuvant, several other approaches are available. In their paper, Xiang et al. describe that polystyrene nanoparticles can induce T cell responses to tumor antigen peptides although not through conventional inflammation. Vermaelen gives an overview of the adjuvant formulations that have been developed to unlock clinically relevant immune responses against cancer antigens, which comprise both immune stimulation and suppressing the suppressors. However, a reality check of the vaccine formulations tested clinically in lung cancer shows that clinical successes are limited and that traditional approaches from the infectious diseases' vaccine field cannot be translated to cancer treatment as such. Ho et al. also report recent insights in clinically relevant vaccine adjuvants that impact DC cross-presentation efficiency. Furthermore, they emphasize that the mode of action of adjuvants in general, and on antigen cross-presentation in DCs in particular, is important for the design of novel adjuvants as part of vaccines able to induce strong cellular immunity. Kartikasari et al. describe the epigenetic effects of vaccine adjuvants on immune cells and cancer cells and propose epigenetic interventions that could improve cancer vaccines.

Another crucial component for the induction of a successful anti-tumor response is the use or targeting of the right **antigen-presenting cell**. DCs are the most professional antigen-presenting cells but, even between the different DC subsets significant functional differences have been reported (4). The review by Clappaert et al. provides a nice overview of the different myeloid cell types that are present in tumors, including DCs, and how they can be harnessed for cancer therapy. Since efficient cross-presentation of tumor antigens is warranted, the current evidence points toward the **cross-presenting DC** subset (CD141^+^ DC in humans, CD8α^+^/CD103^+^ DC in mice) as the most promising target, which is discussed by Mastelic-Gavillet et al. and Ho et al. In this respect, Botelho et al. show specific binding and uptake of a fusion protein of Xcl1 and OVA synthetic long peptide (SLP) by Xcr1^+^ DCs. The potent adjuvant effect on the induced T cell response was associated with sustained tumor control. Thus, developing Xcl1-SLP-Fc fusion proteins as an off-the-shelf vaccine targeting cross-presenting DCs might be an economical and easier alternative to *ex vivo* DC vaccines. Viral vectors constitute another approach to modify DCs *in situ*, as discussed by Goyvaerts and Breckpot. Their attractiveness lies in the fact that they can be targeted and then simultaneously deliver the encoded tumor antigen to antigen-presenting cells as well as behaving as Th1-polarizing adjuvant via the viral vector backbone. However, the antiviral immunogenicity also carries their weakness for which solutions are discussed.

DC targeting can also be achieved via so-called ***in situ* vaccination** approaches, to induce local release of tumor antigens from the tumor itself (5). Yasmin-Karim et al. report that stereotactic body radiation therapy (SBRT) synergizes with intratumoral injection of agonistic anti-CD40, resulting in regression of non-treated contralateral tumors and formation of long-term immunologic memory in a pancreatic mouse model. Locy et al. discuss how oncolytic viruses, radiotherapy, physical therapies, growth factors, and cytokines can stimulate anti-tumor immune responses through the induction of immunogenic cell death, the attraction of different immune cell populations and by alleviating immune suppression. Next challenges for *in situ* vaccination include the accessibility of the tumor and the need to develop approaches to circumvent local immunosuppression.

## Future Perspectives

Although it has come a long way, there is still a lot of room for cancer vaccine optimization. First, the best vaccination approach might differ for “hot tumors (immunogenic)” vs. “cold tumors (non-immunogenic).” Vermaelen describes the importance to focus on **lymphocyte entrance and the local suppression** in the tumor mediated by receptors/ligands (checkpoints), cells (Treg, MDSC), and metabolism (IDO, adenosine, lack of arginine, etc). Strategies to handle tumor associated myeloid cells are more extensively elaborated by Clappaert et al.

Second, **biomarkers** can guide physicians in their treatment decision to obtain a faster selection of the most effective treatment. Highly reliable molecular and/or cellular biomarkers for vaccine efficacy are still to be identified. Mastelic-Gavillet et al. summarizes that in non-small cell lung cancer BDCA1^+^ DC/BDCA3^+^ DC ratio in peripheral blood correlated with survival, as did CD56^dim^ cytotoxic NK cells in glioblastoma. The expression of chemokine receptor CXCR4 on CD8^+^ T cells and CD32 on monocytes correlated with immunological responders. However, these still require further validation. Epigenetic mapping could be a promising next type of biomarker, but is still in its infancy according to Kartikasari et al.

Finally, the indication for which the vaccine developed is of major importance. Due to the highly immunosuppressive nature of the tumor microenvironment, it is clear that cancer vaccination strategies will have to be integrated in **combination therapies** to tackle tumor-induced immunosuppression (6). Current standard of care therapies can have immune modulating properties or serve as adjuvant. Some are described by Locy et al., as mentioned above. Klausen et al. mentions upregulation of cancer testis antigens by hypomethylating agents given to patients with high-risk myelodysplastic syndrome. Practically, the influence of different standards of care in each indication need to be taken into account to foster clinical implementation, in particular when vaccination would not be applied as a first line treatment. Equally important, is looking at the development of new therapies in that indication that might become the next standard of care and existing therapies for other indications that can serve as good adjuvants as mentioned by Ho et al. The review paper of van Willigen et al. delineates the position of DC therapy in the current and future cancer treatment landscape for glioblastoma, melanoma, prostate cancer, and renal cell carcinoma.

**Personalization**, as indicated in this Research Topic, either through the *in situ* or *ex vivo* use of the right type of autologous cell and/or by choosing the best specific target for each tumor or its microenvironment currently holds a lot of promise. Optimized clinical trials will now have to reveal whether this brings cancer vaccine efficacy to the next level.

## Author Contributions

All authors listed have made a substantial, direct and intellectual contribution to the work, and approved it for publication. AV and ST wrote the manuscript. CB performed critical revision.

### Conflict of Interest Statement

The authors declare that the research was conducted in the absence of any commercial or financial relationships that could be construed as a potential conflict of interest.
